# Health- related quality of life in diabetic people with different vascular risk

**DOI:** 10.1186/1471-2458-12-812

**Published:** 2012-09-20

**Authors:** Juan Oliva, Antonio Fernández-Bolaños, Álvaro Hidalgo

**Affiliations:** 1 Economic Analysis Department, University of Castilla-La Mancha (Spain), Facultad de CC. Jurídicas y Sociales de Toledo, Cobertizo de San Pedro Mártir, s/n, 45071, Toledo, Spain

## Abstract

**Background:**

The number of papers on the health related quality of life of patients with DM has grown in recent years but fewer studies have drawn comparisons between diabetic persons and the general population considering different risk groups. The aim of this study is to examine health related quality of life (HRQOL) in people with diabetes mellitus (DM) and to analyze the differences in HRQOL adjusting by vascular risk.

**Methods:**

The data used in this analysis was obtained from the responses of 15,926 individuals who participated in the 2006 Catalonia Health Survey. Our analysis provides a number of multivariate statistical models designed for studying HRQOL, based on the EQ-5D questionnaire, controlling for demographic factors of survey participants and variables that identify diagnosed illnesses and health problems.

**Results:**

Our findings suggest there is a significant, moderate negative relationship between DM and HRQOL in comparison with non diabetic people (absolute value of the coefficient ranges between −0.04 and −0.054 points on a scale of 1). A further analysis of subgroups reveals that diabetics who have not had vascular risk factors neither vascular diseases do not have a diminished HRQOL when compared to the non-diabetic population in general, when other factors are controlled for. In contrast, a comparison of diabetics and non-diabetics who exhibit vascular disease or risk factors for vascular disease reveals HRQOL is significantly diminished to a greater extent for those with diabetes (between 0.152 and 0.175 points loss when comparing a non-diabetic person with a diabetic with vascular disease). Also, HRQOL in diabetic patients who have additional risk factors or a vascular disease are lower than people non-diabetic who have additional risk factors or a vascular disease. When we focus our analysis to the EQ-5D dimensions, we observe that diabetic persons who are neither at risk for nor have a diagnosed vascular disease are no more likely than non-diabetics to report problems. However, diabetic patients who have additional risk factors for vascular disease or a diagnosed vascular disease are significantly more likely to report moderated or severe problems in 4 of the 5 dimensions of EQ-5D.

**Conclusions:**

The HRQOL of a person who has diabetes is not necessarily lower than for a non-diabetic. Control of risk factors associated to vascular diseases is a key factor for an enhanced quality of life. Vascular disease or risk factors for vascular disease, on the other hand, are associated with a significantly diminished quality of life for diabetic persons.

## Background

DM, whether Type 1 or Type 2, is a chronic disease with a high prevalence rate among the world’s population that increases mortality of the people who suffer from it [1]. Within a global framework, it has been estimated that the number of persons with DM in 2000 amounted to 171 million. This number is predicted to reach 366 million by 2030 [2], with 90% of the cases being Type 2. According to the World Health Organization [3], this disease causes 3.2 million deaths a year when deaths from other causes attributable to complications from diabetes are included and it generates substantial health and social welfare costs [4-7].

A person with diabetes may have medical problems caused by acute complications of the disease, such as diabetic ketoacidosis or hyperglycemic hyperosmolar, or as a result of adverse effects of medication, causing for example, cases of hypoglycemia. However, there are chronic complications related to diabetes that increase a diabetic person’s chances of premature death from diseases caused by diabetes, which may be cardiovascular, cerebrovascular, or affect the kidneys [8,9]. Moreover, the quality of life of patients who have diabetes is diminished as a result of the aforementioned problems, and others, such as loss of vision due to diabetic retinopathy or amputations due to peripheral vascular diseases or neuropathy, among other problems [10,11].

Given the complications that DM poses insofar as it encroaches on a person's ability to live a life without limitations as well as the significant rise in the mortality rate of this disease and/or complications arising therefrom, a study on how diabetes mellitus affects the lives of people who suffer from it is of great interest. The number of papers on the quality of life of patients with DM has grown in recent years; the majority have been studies limited to adult populations with Type 2 diabetes. Far fewer studies have drawn comparisons between diabetic persons and the general population or made use of the data from general health surveys in order to compare different risk groups [12-22]. The main objectives of this study are to analyze the HRQOL of patients with DM, compare their situation to that of the general population by using data from the general health survey, and to compare the HRQOL of different risk groups.

## Methods

The data used in the analysis of health-related quality of life were obtained from the 2006 Health Survey of Catalonia (CHS), which was used to gather information on the general health of non-institutionalized adults (16 or more years of age). The CHS microdata were provided to the authors by the Department of Health of the Generalitat (Government) of Catalonia. Microdata are provided for research purposes with the only requirement to complete an application form that explains the aim of the research.

Participants in the survey spent most of the year residing in family dwellings that were their habitual residences. Individuals were excluded if they resided in group homes or were hospitalized at the time of the survey. The CHS is representative for each one of the 37 health areas existing in Catalonia with a maximum estimation error of ± 5%. The CHS was made jointly by the Department of Health and the Catalan Institute of Statistics. A random multistage stratified sample was obtained with two stages, first health region and second municipality. The CHS is also representative of regional populations by sex and age groups and it was conducted by specialized interviewers through personal interviews. Individuals interviewed were selected by simple random extraction process without replacement of the Population Register of Catalonia. To avoid sample loss between the theoretical and effective people interviewed five people were selected for each person interviewed for possible substitution according to a strict protocol to replace losses [23]. Adult responses were obtained from 15,926 persons.

The ESCA used the EQ-5D, a well-known generic HRQOL instrument, which consists of five dimensions: mobility, self- care, usual activities, pain/discomfort and anxiety/depression. HRQOL is measured on three levels in regard to functional state (no health problems, some health problems and extreme health problems), resulting in 243 aggregate combinations. Participants were surveyed on the five dimensions of EQ-5D and each observation was translated to a single health score using the Spanish time trade-off (TTO) value set [24,25]. The Spanish value set have scores ranging from −0.653 to 1, where 1 corresponds to a perfect state of health and 0 corresponds to death.

CHS also provides additional variables that could be associated with health-related quality of life: socio-demographic factors (age, gender, level of educational), previously diagnosed diseases or chronic conditions (vascular disease, rheumatic disease, digestive diseases, mental illness, respiratory disease, diabetes mellitus, musculoskeletal diseases), risk factors (hypertension and hypercholesterolemia) and negative health experiences (undergoing hospitalization) and lifestyle (smoking, alcohol intake).

Given the nature of the dependent variable, we perform a multivariate analysis for identifying variables for predicting HRQOL scores reported by the health survey participants. Our empirical strategy starts with a basic model construct (**Model 1**) that uses control variables such as age, sex, previously diagnosed disease, and health problems (diabetes mellitus, risk of vascular disease, vascular disease, musculoskeletal disease, digestive disease, mental illness, other diseases, and report of an accident in the last 12 months). The variable “risk of vascular disease” receives a score of 1 if the person is obese (BMI ≥ 30 kg/m^2^), has hypertension or abnormal cholesterol levels; otherwise the score is 0.

As an abundant literature indicates, hypertension, obesity and hypercholesterolemia represent a higher vascular risk, for both non-diabetic population and for people with diabetes [26-34]. Although we have no clinical measures on levels of blood pressure or cholesterol, data collected provide information on whether a person has been diagnosed with hypertension or hypercholesterolemia and self referred height and weight. The inclusion of this variable can provide interesting results on the influence of these factors of vascular risk in the QOL as other studies have shown [35,36].

The variable “vascular disease” is assigned a score of 1 if the respondent has been diagnosed with or has ischemic heart disease or has had an embolism. Data on other less common vascular diseases has not been collected in this survey. **Model 2**, which is built upon Model 1, adds a host of demographic factors such as marital status and level of education. **Model 3**, in turn, provides additional data on lifestyle (excessive drinking, smoking, illegal drug use).

Due to the continuous nature of the dependent variable, we performed regression models with heteroskedasticity robust least squares estimate by applying the Eicker-White Heteroskedasticity Consistent Covariance Matrix Estimate [37] so that the inference procedures are valid even where the error terms have non constant variance among survey participants.

Furthermore, the two-fold objective of this study led us to construct two versions of each model. In the first version (A), previously diagnosed DM is used as an explanatory variable in the multivariate analysis on the dependent variable where we contrast if diagnosed DM is associated with a lower health-related quality of life. The second version (B) identifies persons who have been diagnosed of diabetes but have neither had not previously been diagnosed with any other vascular disease (ischemic heart disease or embolism) and do not exhibit any reported risk factors (obesity, high cholesterol, hypertension) and compares them to diabetic patients who are at risk for vascular disease and those who have had or have been diagnosed with vascular disease. In addition, control and comparison variables have been introduced by including data on persons who do not have diabetes but are at risk for vascular disease and others who do not have diabetes but have had or have previously been diagnosed with vascular disease. So, Model B can be interpreted as analysis of subgroups.

Lastly, a detailed analysis is performed on each of the dimensions that comprise the research instrument for assessing quality of life (mobility, self-care, usual activities, pain/discomfort and anxiety/depression). The probability of reporting a problem as moderate or extreme for each of the dimensions of the EQ-5D is therefore analyzed using the same explanatory variables from Model 3 as control variables. Given the nature of each of the variables studied (dichotomous variables that receive a score of 1 if the individual identifies a problem in the dimension under study or a score of 0 where no problem is indicated) the analysis is carried out using discrete choice models of the probit type.

## Results

Tables 1 and 2 show the main characteristics of the sample group under analysis. General population report the pain/discomfort dimension as being the most frequent in terms of moderate/extreme problems (34.02%), with moderate/extreme problems being the least frequent (6.29%) in the self-care dimension. Average age was 47.4 years old. A 49.5% of the sample were men. A 14.7% of the sample had no studies completed, 23.7% had primary school studies, 46.5% secondary school studies and 15.1% tertiary studies. Only 6.40% of the sample population report being diabetic, while osteomuscular problems are the most reported (46.85%). A 10.2% of the sample was diagnosed of a vascular disease and a 37.8% presented vascular risk.

**Table 1:** Main characteristics of the sample

**Independent variables**	**Percentage/ average (standard desviation)**
Age	47.38 (19.55)
Male/Female	49.5%/50.5%
**Education**	
No studies completed	14.73%
Primary school completed	23.66%
Secondary school completed	46.50%
University completed	15.11%
**Marital status**	
Singles	30.47%
Married / partnered	57.06%
Widow	8.28%
Separate	4.20%
**Reported diseases**	
Diabetes Mellitus	6.40%
Cardiovascular Risk	37.78%
Vascular problems	10.15%
Osteomuscular problems	46.85%
Respiratory problems	10.17%
Digestive problems	12.27%
Mental illness (anxiety/depression)	18.72%
**Reported health risks**	
Risk alcohol intake	4.63%
Smoker	28.25%
Ex-smoker	19.40%
lllegal Drug user	8.39%

**Table 2:** Distribution of dependent variables

**Dependent variables**	**Percentage/ average (standard desviation)**
EQ-5D score	0.857 (0.264)
Moderate or extreme problem in EQ-5D dimension 1 (Mobility)	16.96%
Moderate or extreme problem in EQ-5D dimension 2 (Self-care)	6.29%
Moderate or extreme problem in EQ-5D dimension 3 (Usual activities)	12.96%
Moderate or extreme problem in EQ-5D dimension 4 (Pain/discomfort)	34.02%
Moderate or extreme problem in EQ-5D dimension 5 (Anxiety/depression)	20.21%

The EQ-5D average score of the sample is 0.857 (with a standard deviation of 0.264). A 16.96% of the sample reported moderate or severe problems in the dimension of mobility. A 6.29% in the dimension of self-care. A 12.96% in the dimension of usual activities. A 34.02% in the dimension of pain/discomfort. And, finally, a 20.21% of the sample reported moderate or severe problems in the dimension of anxiety/depression.

Results of the empirical analysis are contained in Tables 3 and 4. If we focus our attention on the Type A models, we observe that diabetes mellitus is a significant variable that has a negative relationship with health-related quality of life. The absolute value of the coefficient ranges between 0.04 and 0.054 points on a scale of 1 which indicates a moderately diminished HRQOL compared to non-diabetic persons after controlling for other factors. Risk factors for vascular disease are similarly associated with a significant drop in HRQOL, although the absolute value of the coefficient is low. Lastly, the variable “previously diagnosed vascular disease” is clearly significant. It has a negative score and its absolute value exceeds that of the variable “diabetes”, as it falls between 0.076 and 0.1, on a scale of 1, compared with persons who do not have this disease, after controlling for other factors. Results for the Type A models are fairly stable and indicate that DM is associated to a moderate drop in HRQOL. The loss of HRQOL in persons with DM is less than for persons who have vascular disease and greater than the loss of HRQOL when other risk factors are present.

**Table 3:** Health related quality of life

	**Model 1**	**Model 1**	**Model 2**	**Model 2**	**Model 3**	**Model 3**
	**Version A**	**Version B**	**Version A**	**Version B**	**Version A**	**Version B**
**Diabetes mellitus**	−0.0542		−0.0406		−0.0458	
	(0.0102) ***		(0.0099) ***		(0.0094) ***	
**Risk factors for vascular disease**	−0.0105		−0.0056		−0.0116	
	(0.0041) ***		(0.0040)		(0.0037) ***	
**Vascular disease**	−0.1017		−0.0851		−0.0756	
	(0.0094) ***		(0.0090) ***		(0.0083) ***	
**Diabetes without additional risk factors or vascular disease**		−0.0279		−0.0179		−0.0192
		(0.0176)		(0.0178)		(0.0160)
**Diabetes with risk factors for vascular disease**		−0.0595		−0.0496		−0.0566
		(0.0127) ***		(0.0126) ***		(0.0119) ***
**Risk factors for vascular disease without diabetes**		−0.0104		−0.0066		−0.0128
		(0.0041) **		(0.0041)		(0.0038) ***
**Diabetes with vascular disease**		−0.1751		−0.1516		−0.1535
		(0.0217) ***		(0.0209) ***		(0.0211) ***
**Vascular disease without diabetes**		−0.0959		−0.0870		−0.0733
		(0.0100) ***		(0.0099) ***		(0.0089) ***
**Sample size**	15,926	15,926	15,919	15,919	15,455	15,455
**F Distribution**	360.06	315.45	207.92	195.07	162.13	154.11
**R-squared (R**^**2**^**)**	0.3857	0.386	0.4165	0.4041	0.3913	0.3831

*** Significant variable 99% CI ** Significant variable 95% CI.

Model 1: control variables: sex, age, diseases; Model 2: control variables: sex, age, diseases, marital status, level of education, Model 3: control variables: sex, age, diseases, marital status, level of education, alcohol intake, smoking habit, illegal drug use.

Version B of the Models. Reference group characteristics: Non-diabetic, no risk factors for vascular disease, and no diagnosed vascular disease.

Marginal Effects from Empirical Models.

**Table 4:** Health related quality of life by dimensions

	**Dimension 1 ** **Mobility**	**Dimension 2 ** **Self-care**	**Dimension 3 ** **Usual activities**	**Dimension 4 ** **Pain/discomfort**	**Dimension 5 ** **Anxiety/Depression**
**Diabetes without additional risk factors or vascular disease**	0.0201	0.0181	0.0492	0.0775	0.0242
	(0.0234)	(0.0143)	(0.0244)	(0.0429)	(0.0332)
**Diabetes with risk factors for vascular disease**	0.1017	0.0211	0.0454	0.0916	0.0267
	(0.0177) ***	(0.0076) ***	(0.0125) ***	(0.0252) ***	(0.0181)
**Risk factors for vascular disease without diabetes**	0.0404	0.0064	0.0141	0.0470	0.0137
	(0.0063) ***	(0.0029) **	(0.0047) ***	(0.0104) ***	(0.0078)
**Diabetes with vascular disease**	0.1440	0.0519	0.1041	0.1224	0.0357
	(0.0259) ***	(0.0133) ***	(0.0208) ***	(0.0352) ***	(0.0237)
**Vascular disease without diabetes**	0.0844	0.0269	0.0724	0.1189	0.0435
	(0.0121) ***	(0.0061) ***	(0.0105) ***	(0.0184) ***	(0.0136) ***
**Sample size**	15,455	15,455	15,455	15,455	15,455
**F Distribution**	4467.4	1487.73	3259.74	5570.5	4688.82
**Pseudo R**^**2**^	0.3348	0.2529	0.301	0.2839	0.3086

*** Significant variable 99% CI. ** Significant variable 95% CI.

Reference group characteristics: Non-diabetic, no risk factors for vascular disease, and no diagnosed vascular disease.

Control variables: sex, age, diseases, marital status, level of education, alcohol intake, smoking habit, illegal drug use.

Marginal Effects from Probit Models.

The Type B models allow us to compare the HRQOL of persons with DM based on the existence of other risk factors for vascular disease and in cases where vascular disease is or is not present. The results indicate that persons who have diabetes who are not at-risk for vascular disease and have not previously been diagnosed with vascular disease do not have a diminished HRQOL when compared to the rest of the population, after controlling for other factors (age, sex, other diseases, sociodemographic variables and lifestyles). Although the coefficient has a negative value, it is nearly zero and is not statistically significant.

However, in the case of a diabetic person who has additional risk factors for vascular disease, HRQOL drops significantly when compared to a non-diabetic who is not at-risk (a loss of 0.05-0.06 points). Similarly, a comparison of the loss of HRQOL in a diabetic patient who has additional risk factors for vascular disease and a non-diabetic who has additional risk factors for vascular disease, the loss in QOL is significantly greater for persons who have diabetes. The loss of HRQOL is clearly the greatest for diabetic patients who have been diagnosed with vascular disease. Compared to non-diabetic persons that have not been diagnosed with a disease of this type, the loss of HRQOL is statistically significant in all three Type B models (between 0.152 and 0.175 points loss). It is also significant that HRQOL of this group is also lower than for non-diabetic group who had been diagnosed with vascular disease.

When we focus our analysis to the EQ-5D dimensions, we observe that diabetic persons who are neither at risk for nor have a diagnosed vascular disease are no more likely than those in the reference group (non-diabetics with no risk factors or previously diagnosed vascular disease) to report problems in dimensions 1 (physical mobility), 2 (self-care) 3 (usual activities) and 4 (pain/discomfort). In contrast, diabetic patients who have additional risk factors for vascular disease (obesity, hypercholesterolemia, hypertension) or a diagnosed vascular disease are significantly more likely to report in each of the 4 dimensions than the individuals in the reference group. Furthermore, diabetic persons who are at-risk for vascular disease report more problems that non-diabetics who are also at-risk, and diabetic persons who have been diagnosed with vascular disease have more problems than non-diabetics who have been diagnosed with vascular disease.

## Discussion and conclusions

Our findings reveal a significant inverse relationship between diabetes mellitus and health related quality of life. We should emphasize that problems are much more pronounced in diabetic population who exhibit additional risk factors for vascular disease and especially in cases when vascular disease is already present. The information provided in our analysis of subgroups is especially valuable for health policy decision makers and could serve as the basis for measures and healthcare policies [38].

The main result of this study is that the health-related quality of life of persons who have diabetes need not be lower than it is for non-diabetic persons. Managing diabetes mellitus by controlling blood glucose levels, blood pressure, cholesterol levels and weight as well as implementing preventative measures to avoid acute vascular events are key factors that can lead to an enhanced quality of life. These results mirror those obtained in studies performed in other countries [12-14,39,40] where diabetic people with healthy habits and less cardiovascular risks report substantially better HRQOL.

However, a key aspect or our study is that our empirical results are not only statistically significant, but given the magnitudes of the negative effect of vascular risk and vascular disease on the quality of life of diabetic people, in comparison with non diabetic people and diabetic people with no additional risks, they are also clinically relevant. The observed changes in HRQOL are much higher than the values often cited in international literature as the minimal clinically important difference where responder definitions for the EQ-5D TTO ranged from 0.074 to 0.08 [41,42]. Results that are statistically and clinically significant might be robust enough to serve as the basis for health policies that could benefit wide population groups. In addition, vascular risk and vascular diseases in diabetic people are associated with significant worsening in the EQ-5D dimensions relating to pain/discomfort, mobility, self-care and usual activities. In contrast, we do not identify statistically significant differences between diabetic and non-diabetic people when it comes to self perceived mental health, after controlling for other factors. This result is consistent with other previous papers [21,39,43].

Certain limitations in this study must be mentioned. Firstly, in spite of the robust set of explanatory variables, the cross-sectional nature of the data does not allow us to perform a longitudinal analysis of the state of health of respondents over a period of time. This is of great importance because the study focuses on the quality of life for the years lived, but diseases of the circulatory system comprise the leading cause of death in Spain and is one of the top 3 causes of years of potential life lost (YPLL). Longitudinal data bases (questionnaires or administrative data bases) allow for an analysis of not only the loss in quality of life but the differences in life expectancy between diabetic and non-diabetic population, while controlling for other risk factors and diagnosed vascular disease, for the purpose of calculating the Adjusted Life years for diabetic and non-diabetic persons [44-46]. Secondly, body mass index (BMI) was calculated based on self-reported data on height and weight provided by survey respondents, thereby making responses susceptible to potential bias inherent in how respondents perceive themselves [47-50]. Similarly, there may be people with diabetes who have not been diagnosed with the disease. Therefore, the conclusions of the analysis should be limited to people who know they are diabetic. Also, it would be interesting to have information on the time since diagnosis of DM, vascular risks or vascular diseases to avoid the confounding effects that cardiovascular risk may introduce in the estimates.

It should be stressed that the CHS doesn’t allow us to perform a separate analysis for people with type 1 and type 2 DM. It is expected that most of the diabetic people involved in the survey present type 2 Diabetes. However, it is not possible to confirm this point. Therefore, the accuracy of the results achieved in this study and the differentiation of the effect of vascular risk and vascular diseases in people with type 1 and type 2 diabetes is a line of research to focus future analysis.

Finally, a further limitation of our analysis is that, when incorporating vascular risk factors to evaluate HRQOL of patients with diabetes, we did it from the data at our disposal from the CHS, which do not follow the pattern of a recognised clinical measure, as the Framingham or SCORE scores. CHS is a general health survey that does not provide us these clinical scores. Data collected provide information on whether a person has been diagnosed with hypertension or hypercholesterolemia and self referred height and weight but not on clinical levels of blood pressure or cholesterol or on the measured height and weight. A future line of research would be to analyze the combined effect of diabetes, hypertension, obesity and hypercholesterolemia on health related quality of life in a group of people where the variables of interest were measured under criteria and clinical parameters.

The main objective of this study was to examine HRQOL in people with DM and to ascertain data that would be important for both individuals who have DM, and healthcare decision makers. This information should prove useful for achieving adequate healthcare for persons with diabetes mellitus, starting off by providing them with the necessary tools and resources for acquiring a better understanding of the significance of their health condition and to become experts on how to handle the disease itself; such a disease management program for type 2 diabetes patients can improve their HRQOL [51]. In time we hope that this information will prove useful to healthcare policy makers, serving as a basis on which to make decisions regarding the efficient allocation of resources.

Within the policy making realm this data could be included in the process to develop a Health Strategy in which the first step would be to provide sufficient epidemiological data for identifying key health problems of the region or country under study, which would include information on the prevalence and incidence of diabetes, potential years of life lost (or gained) and mortality rates, disabilities that were avoided or developed, and gains or losses in HRQOL. Secondly, information about costs associated with DM and related chronic complications (employing a broad concept of costs that includes losses in labour production, social costs associated with the loss of independence, and healthcare spending) could be used as an approximation of the loss in social welfare these diseases cause. The third step would be to have information available on the technical and human resources that could be employed in policies or plans of action that affect primary, secondary and tertiary preventive measures aimed at DM and its complications. After these steps have been taken, a logical move would be to identify programs and measures across different areas that are efficient, namely policies that lead to improved life expectancy and quality of life for those who suffer from these diseases while reducing the burden they pose on available resources. Subsequently, these measures should be put into operation and evaluated.

Thus, a series of health-related measures in various areas (healthcare, education, etc.) that are scientifically proven to be effective and efficient at improving the situation of the population whose health is affected by health problems caused by diabetes mellitus could be an invaluable tool on which to base healthcare policy decisions about the allocation of resources (evidence-based policies). Knowledge of the diminished health-related quality of life in persons with diabetes mellitus should be among the indicators that health policy decision makers consider if they aim to formulate health policies that are efficient from a social perspective.

## Abbreviations

HRQOL: Health-related quality of life; DM: Diabetes Mellitus; EQ-5D: EQ-5D questionnaire; CHS: Catalonia Health Survey; BMI: Body Mass Index; QOL: Quality of Life; YPLL: Years of Potential Life Lost.

## Competing interests

JO, AH and A F-B have received a grant/research support from Novo Nordisk.

## Authors’ contributions

JO designed the study, carried out statistical models, wrote and reviewed manuscript; A F-B searched background, wrote and reviewed manuscript; AH, wrote and reviewed manuscript. All authors read and approved the final manuscript.

## Pre-publication history

The pre-publication history for this paper can be accessed here:

http://www.biomedcentral.com/1471-2458/12/812/prepub
